# Alcohol and trauma: the influence of blood alcohol levels on the severity of injuries and outcome of trauma patients - a retrospective analysis of 6268 patients of the TraumaRegister DGU^®^

**DOI:** 10.1186/s13049-021-00916-z

**Published:** 2021-07-27

**Authors:** Thomas Brockamp, Andreas Böhmer, Rolf Lefering, Bertil Bouillon, Arasch Wafaisade, Manuel Mutschler, Paola Kappel, Matthias Fröhlich, Working Group of Injury Prevention of the German Trauma Society (DGU)

**Affiliations:** 1 Department of Trauma and Orthopaedic Surgery, University of Witten/Herdecke, Cologne-Merheim-Medical-Center, Ostmerheimer Street 200, 51109 Cologne, Germany; 2 Department of Anaesthesiology and Surgical Intensive Care Medicine, University of Witten/Herdecke, Cologne-Merheim-Medical-Center, Ostmerheimer Street 200, 51109 Cologne, Germany; 3 Institute for Research in Operative Medicine (IFOM), University of Witten/Herdecke, Ostmerheimer Street 200, 51109 Cologne, Germany; 4 Working Group of Injury Prevention of the German Trauma Society, The German Trauma Society (DGU), Straße des 17. Juni 106-108, 10623 Berlin, Germany

**Keywords:** Blood alcohol, Traumatic brain injury, Outcome, Youth

## Abstract

**Background:**

Blood alcohol level (BAL) has previously been considered as a factor influencing the outcome of injured patients. Despite the well-known positive correlation between alcohol-influenced traffic participation and the risk of accidents, there is still no clear evidence of a positive correlation between blood alcohol levels and severity of injury. The aim of the study was to analyze data of the TraumaRegister DGU^®^ (TR-DGU), to find out whether the blood alcohol level has an influence on the type and severity of injuries as well as on the outcome of multiple-trauma patients.

**Methods:**

Datasets from 11,842 trauma patients of the TR-DGU from the years 2015 and 2016 were analyzed retrospectively and 6268 patients with a full dataset and an AIS ≥ 3 could be used for evaluation. Two groups were formed for data analysis. A control group with a BAL = 0 ‰ (BAL negative) was compared to an alcohol group with a BAL of ≥0.3‰ to < 4.0‰ (BAL positive). Patients with a BAL >  0‰ and <  0.3‰ were excluded. They were compared with regard to various preclinical, clinical and physiological parameters. Additionally, a subgroup analysis with a focus on patients with a traumatic brain injury (TBI) was performed.

A total of 5271 cases were assigned to the control group and 832 cases to the BAL positive group. 70.3% (3704) of the patients in the control group were male. The collective of the control group was on average 5.7 years older than the patients in the BAL positive group (*p* < .001). The control group showed a mean ISS of 20.3 and the alcohol group of 18.9 (*p* = .007). In terms of the injury severity of head, the BAL positive group was significantly higher on average than the control group (*p* <  0.001), whereas the control group showed a higher AIS to thorax and extremities (*p* <  0.001). The mean Glasgow Coma Scale (GCS) was 10.8 in the BAL positive group and 12.0 in the control group (*p* <  0.001). Physiological parameters such as base excess (BE) and International Normalized Ratio (INR) showed reduced values ​​for the BAL positive group. However, neither the 24-h mortality nor the overall mortality showed a significant difference in either group (*p* = 0.19, *p* = 0.14). In a subgroup analysis, we found that patients with a relevant head injury (AIS: Abbreviated Injury Scale head ≥3) and positive BAL displayed a higher survival rate compared to patients in the control group with isolated TBI (*p* < 0.001).

**Conclusions:**

This retrospective study analyzed the influence of the blood alcohol level in severely injured patients in a large national dataset. BAL positive patients showed worse results with regard to head injuries, the GCS and to some other physiological parameters. Finally, neither the 24-h mortality nor the overall mortality showed a significant difference in either group. Only in a subgroup analysis the mortality rate in BAL negative patients with TBI was significantly higher than the mortality rate of BAL positive patients with TBI. This mechanism is not yet fully understood and is discussed controversially in the literature.

## Background

Every year, 1.2 million people worldwide die in road accidents [1]. Across the EU, more than 30,000 people die each year in road accidents. Millions more suffer injuries and permanent disabilities. Alcohol and drug abuse are often the causes of serious road accidents [2]. In Germany, a total of 2,5 million accidents were recorded by the police in 2018, including 37,450 accidents in which at least one person was under the influence of intoxicating substances. Alcohol influence was one of the causes of accidents in 2017 for 4.1% of all accidents in which persons have been involved. Overall, 7.4% of all fatally injured road users in Germany died as a result of an accident due to alcohol abuse. The number of accidents under the influence of other intoxicating substances increased sharply between 1991 and 2017 and almost quadrupled, from 434 to 1679 accidents [3].

Despite the well-known positive correlation between alcohol-influenced traffic participation and the risk of accidents, there is still no clear evidence of a positive correlation between blood alcohol levels (BAL) and severity of injury. Moreover, it is not clear what precise influence the BAL has on the outcome (mortality; hospital stay, etc.) of a trauma patient injured in a traffic accident. Several studies have shown that patients with acute alcohol intoxication have a shorter hospital stay than patients without alcohol exposure [4–6]. However, other authors could not prove a link between alcohol misuse and the severity of injury or outcome [7, 8] or show that alcohol consumption increases the severity of injury [9]. The reasons for these different results can certainly also be found in the retrospective study designs and applied methodologies. Nevertheless, it is important to further analyze the effects of alcohol and drugs on the human body in the context of a seriously injured person in order to understand existing mechanisms of action. We think that our work can help to underline the important relation between the influence of alcohol on the body in multiple trauma patients. It is not yet sufficiently understood how exactly alcohol changes the physiology of different systems in the human body (e.g. brain; blood; coagulopathy; blood pressure). To our knowledge, the literature does not give an adequate answer and in vitro studies on multiple trauma patients with a measurable blood alcohol level are rare.

The aim of the present study was to check whether the blood alcohol level (measured in permille) has an influence on the nature and severity of injuries as well as on the outcome of trauma patients. A retrospective analysis using data of the TR-DGU was performed.

## Methods

In this retrospective observational study, data of 11,842 trauma patients from the TraumaRegister DGU®, collected between 2015 and 2016, were analyzed. The alcohol parameter was included in the registry in 2015. 6103 data sets were used for data analysis. Only primary admitted patients with an AIS ≥ 3 for whom a measured blood alcohol level was documented, were included. Patients transferred out early (< 48 h) to another hospital (missing outcome) or patients who were secondary admitted (missing initial BAL) were not included.

Two groups were formed for data analysis. A control group with a BAL = 0 ‰ (BAL negative) and an alcohol group with a BAL of ≥0.3‰ to < 4.0‰ (BAL positive). Values of 4‰ or higher were considered unreliable and therefore excluded. The lower limit value (0.3‰) was chosen because there are already measurable effects on the body. Patients with a BAL >  0‰ and < 0.3‰ were excluded. Additionally, a subgroup analysis was done to investigate the effect of alcohol in a dataset of patients with a severe head injury (AIS head ≥3).

Preclinical, clinical and physiological parameters were compared, as were different outcome parameters between the two groups. Differences between the groups were analyzed at a significance level *p* < 0.05 using the Mann-Whitney-U and Pearson’s Chi-Squared test when appropriate. The data is presented as mean with standard deviation for continuous variables and as a percentage for categorical variables. The statistical analysis was performed using standard statistics software (SPSS version 24.0, IBM, Armonk, NY, USA).

### TraumaRegister DGU®

The TraumaRegister DGU^®^ of the German Society of Trauma Surgery (DGU) was founded in 1993. The aim of this multicentre database is to provide pseudonymised and standardised documentation of seriously injured persons.

The data are collected prospectively in four successive phases: A) preclinical phase, B) shock room and subsequent surgical phase, C) intensive care unit and D) discharge. The documentation contains detailed information on demography, injury patterns, comorbidities, preclinical and clinical management, intensive medical history, important laboratory findings including transfusion data, and outcome. The inclusion criterion is admission to the hospital via the shock room followed by intensive or intermediate care unit monitoring, or arrival in the clinic with vital signs and death before admission to the intensive care unit.

The infrastructure for documentation, data management and data analysis is provided by the AUC - Akademie der Unfallchirurgie GmbH, which is affiliated with the DGU.

The scientific leadership is provided by the Committee on Emergency Medicine, Intensive.

Care and Trauma Management (Sektion NIS) of the German Trauma Society. Through a web-based application, the participating clinics enter their data pseudonymously into a central database. Scientific evaluations are approved according to a review procedure of the NIS section. The participating clinics are primarily located in Germany (90%) but an increasing number of clinics from other countries also contribute data (currently from Austria, Belgium, China, Finland, Luxembourg, Slovenia, Switzerland, the Netherlands and the United Arab Emirates). Currently, about 33,000 cases of more than 650 clinics are included in the database each year.

This study complies with the publication guidelines of the TraumaRegister DGU^®^ and is registered as TR-DGU Project ID 2017–020 N.

## Results

A total of 5271 cases were assigned to the control group and 832 cases to the BAL positive group. Basis study characteristics are provided in Table 1. It shows that 70% of the patients in the control group were male. The collective of the control group was on average 6 years older than the patients in the BAL positive group (*p* < .001). The evaluation of the physiological parameters showed a higher blood pressure level in the control group (133 versus 125 mmHg) and a low hemoglobin value (12.9 versus 13.4). Base excess showed significantly lower levels in the BAL positive group. With regard to the ISS, the control group shows an average value of 20.3 and the alcohol group of 18.9 (*p* = .006). In addition, the average GCS was 10.8 in the BAL positive group and 12.1 in the control group (p < .001).

**Table 1:** Patient characteristic

		BAL negative	BAL positive	*p*-value
Male	n (%)	3704 (70.3)	699 (84.0)	<.001
Age (years)	(Mean, SD)	51 ± 22	45 ± 18	<.001
GCS	(Mean, SD)	12.0 ± 4.1	10.8 ± 4.5	<.001
systBP on admission	(Mean, SD)	133 ± 32	125 ± 29	<.001
Hemoglobin (g/dl)	(Mean, SD)	12.9 ± 2.2	13.4 ± 2.1	<.001
INR	(Mean, SD)	1.20 ± 0.54	1.13 ± 0.49	<.001
BE (mmol/l)	(Mean, SD)	−1.8 ± 4.4	−3.9 ± 4.5	<.001
ISS	(Mean, SD)	20.3 ± 12.6	18.9 ± 11.6	.006
NISS	(Mean, SD)	25.3 ± 15.3	24.9 ± 15.9	.27

BAL, Blood Alcohol Levels; systBP, Systolic Blood Pressure; GCS, Glasgow Coma Scale; INR, International Normalized Ratio; BE, Base Excess; ISS, Injury Severity Score; NISS, New Injury Severity Score; SD Standard Deviation

Regarding the injury severity in different body regions, we found significantly higher values regarding AIS head of BAL positive patients (*p* < 0.001) whereas AIS thorax and AIS extremities were significantly lower in the BAL positive group compared to the control group (p < 0.001). No significant differences could be measured for AIS abdomen (Table 2).

**Table 2:** AIS depending on BA

Relevant injury (AIS ≥ 3) of		BAL negative	BAL positive	*p*-value
Head	n (%)	2092 (39.7%)	405 (48.7%)	<.001
Thorax	n (%)	2195 (41.6%)	267 (32.1%)	<.001
Abdomen	n (%)	570 (10.8%)	77 (9.3%)	.18
Extremities / pelvis	n (%)	1378 (26.1%)	143 (17.2%)	<.001

BAL, Blood Alcohol Levels; AIS, Abbreviated Injury Scale

Table 3 gives an overview of the type of accident depending on BAL. A significantly increased proportion of BAL positive patients suffered from low falls, while mechanisms with increased force, such as car or motorcycle accidents, were less common in this group (*p* < 0.001).

**Table 3:** Mechanism of Injury according to BAL

		BAL negative	BAL positive	*p*-value
Car	n (%)	1256 (23%)	122 (17%)	> 0.05
Motorbike	n (%)	769 (14%)	53 (6%)	> 0.05
Bicycle	n (%)	519 (9%)	70 (8%)	> 0.05
Pedestrian	n (%)	366 (7%)	76 (9%)	> 0.05
Fall from > 3 m	n (%)	913 (17%)	126 (15%)	> 0.05
Fall from < 3 m	n (%)	963 (18%)	262 (30%)	< 0.001
Blunt trauma	n (%)	5050 (97%)	781 (93%)	< 0.001
Penetrating trauma	n (%)	107 (2%)	44 (5%)	> 0.05

BAL, Blood Alcohol Levels

Neither the 24-h mortality nor the overall mortality showed a significant difference in either group (*p* = 0.19, *p* = 0.14). No relevant differences were found for ventilation days and length of stay on intensive care unit (ICU) or length of stay in hospital (Table 4).

**Table 4:** Outcome according to BAL

		BAL negative	BAL positive	*p*-value
Mechanical ventilation (days)	Median (IQR)	0 (0–3)	0 (0–2)	.13
LOS on ICU (days)	Median (IQR)	3 (1–9)	2 (1–8)	.23
LOS in hospital (days)	Median (IQR)	13 (7–23)	12 (6–23)	.026
Mortality (24 h)	n (%)	266 (5.0%)	33 (4.0%)	.19
Mortality (in hospital)	n (%)	654 (12.4%)	88 (10.6%)	.14
95% CI for Mortality		[11.5–13.3]	[8.5–12.7]	
RISC II Prognose		11.7%	10.8%	.97
**Subgroup TBI**	n	2092	405	
ISS	Mean (SD)	27 (13)	24 (12)	<.001
Isolated TBI	n (%)	653 (31.2%)	173 (42.7%)	<.001
Mortality (in hospital)	n (%)	509 (24.3%)	68 (16.8%)	.001
95% CI for mortality		[22.5–26.2]	[13.1–20.4]	
RISC II prognosis	Mean	23.9%	18.7%	<.001

LOS, Length of stay. ICU, Intensive Care Unit; TBI, Traumatic brain injury; CI Confidence Interval

We further analyzed the influence of alcohol on trauma patients with a combination of a severe TBI (AIS head ≥3) and a positive BAL. This subgroup analysis showed that the mortality rate in patients with a positive BAL and a severe TBI was significantly lower (16.8%) compared to patients of the BAL negative group (24.3%) (*p* = .001) although the BAL positive group showed more isolated traumatic brain injuries (43% versus 31%, *p* < .001). The ISS showed higher values in the BAL negative group (27 vs. 24, *p* < 0.001).

The Revised Injury Severity Classification (RISC II) Score was additionally used to compare the observed with the predicted mortality. However, predicted mortality differed by less than 2% from observed mortality in both groups. In the subgroup TBI observed mortality was about 2% better than predicted (BAL positive), but the 95% CI was too broad to prove this effect.

**Fig. 1:**
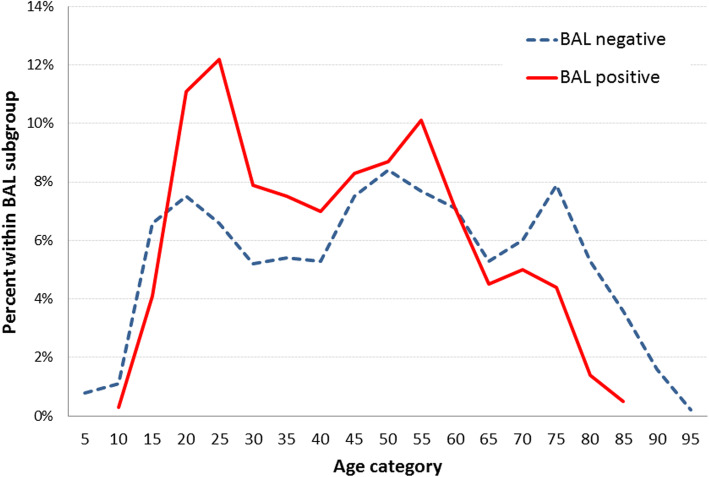
Age distribution of patients with (*n* = 832) and without (*n* = 5271) positive BAL

Figure 1 shows the relation between age and BAL positive and BAL negative patients. Younger patients (< 15 years) as well as older patients (> 60 years) were more frequently found in the control group, whereas patients about 25 and 55 years show an increased proportion in the BAL positive group.

## Discussion

The influence of blood alcohol on the human body has been described many times [10–12]. In addition to the negative effects on, among others, liver function or the central nervous system caused by ethanol, there are studies that describe a protective effect on some aspects, such as the cardiovascular system [13]. Road accidents often occur under the influence of alcohol and drugs. The risk of a road accident increases with increasing inability to drive, caused by alcohol and drugs [14, 15]. However, it is not fully understood what effect a positive BAL has on the physiological parameters and outcome of a seriously injured patient. For example, some authors describe that they measured a lower GCS and lower blood pressure parameters in patients with positive BAL compared to BAL negative patients, and finally observed an increased mortality rate for BAL positive patients [9]. A positive BAL can also affect the occurrence of a traumatic brain injury (TBI). In animal models, alcohol has shown both adverse and neuroprotective effects, while clinical studies analyzing acute and long-term neurological and behavioral effects of intoxication show no consistent results [4, 16, 17]. Our study results confirm some of the hypotheses described. The majority of our study collective consists of young, male patients. This coincides with the characterization of the risk profile of injured, alcoholized patients that can also be found in other studies [18, 19].

First, we found no significant differences in the 24 h mortality. The overall mortality rate showed no significant difference between the groups. Furthermore, the RISC II analysis confirmed our findings (predicted mortality). Looking at all patients in our study collective, no differences in mortality were seen. At most, the length of stay in the hospital was lower in patients with a positive BAL.

We then performed a subgroup analysis to focus on the group of patients with a severe traumatic brain injury (AIS head ≥3) to look for further effects that might show different results. However, we showed that the mortality rate in BAL positive patients with TBI was significantly lower than the mortality rate in BAL negative patients with TBI. Additionally, in our subgroup analysis, we observed more patients with an isolated TBI. But the ISS was lower in the BAL positive group. The ISS might have an effect on the higher mortality rate in the BAL negative subgroup.

Age might be influencing the outcome of mortality in this subgroup, too. But we have the same significant age difference when testing for 24 h mortality and overall mortality in the whole collective. Thus, we do not think that age might be one of the main influencing factors.

Some research groups also found a positive, protective effect of ethanol in patients with moderate and severe TBIs. They showed a significantly lower mortality rate for this group [20].

Tien et al. showed that low to moderate BAL can be beneficial in patients with severe TBI but a high BAL appears to cause an increased risk of mortality during a hospital stay in these patients, which may be related to the adverse hemodynamic and physiological effects in BAL positive patients [21]. Sperry et al. investigated the influence of alcohol on GCS in TBI patients. They showed that positive BAL in patients with blunt TBI does not lead to clinically significant changes in GCS value [22]. On the other hand, Pandit et al. reported that patients with severe TBI and positive BAL more frequently develop complications during a hospital stay compared to BAL negative patients with TBI. They were therefore unable to detect any neuroprotective effect related to ethanol [23].

In conclusion, a neuro-protective effect of alcohol and the significant survival benefit for alcohol intoxicated patients with a traumatic brain injury [20, 24] cannot be clearly explained and remains of further interest.

In the BAL positive group, a change in physiological parameters such as blood pressure and BE was observed. Alcohol has complex effects on the circulatory system. The relationship between alcohol and high blood pressure is well known, but little is known about the effect of alcohol on the circulation in seriously injured patients. Our results show a small drop in blood pressure in patients in the alcohol group. These results are in line with those of other authors [5, 25]. In clinical trials, patients with an additional traumatic brain injury had lower amounts of catecholamines in the bloodstream, which in turn resulted in lower systolic blood pressure when admitted to the hospital [26–28].

Patients with a positive BAL have a lower base excess than those in the BAL negative group.

Since alcohol consumption itself leads to lower values of BE (or higher values of lactate), it is questionable whether these markers could be used as a screening tool for injured intoxicated patients. Normally, the BE is an indicator for tissue hypoperfusion. However, according to a study by Dunne et al., the positive significance of the base excess as a predictive value in relation to mortality remains regarding BAL positive patients [29].

Other studies show a comparable influence on various physiological parameters, in particular on base excess and lactate value, without measuring a difference in mortality [29].

The influence of alcohol on the coagulation system is controversial. In connection with traumatic brain injuries, many studies suggest that with the simultaneous presence of traumatic brain injury and a blood-increased alcohol level, the clotting system is affected. For example, Howard et al. showed that patients with brain injury and positive BAL had a significantly lower incidence of coagulopathies [30]. Since we did not focus on coagulopathies, we cannot make any further reliable statements based on our data but the effect remains of certain interest especially in trauma care.

In our analysis, patients in the alcohol group show an increased risk of falling from a low altitude (< 3 m) compared to patients in the control group. However, we have not been able to prove that patients from the alcohol group are more likely to be involved in accidents with e.g. cars, motorcycles or bicycles. This is likely due to the fact that the majority of BAL positive people no longer drive their own cars, motorbikes or bicycles, but rely on taxis or public transport. The increased number of low falls with an increased rate of severe traumatic brain injuries may be attributed to the fact that the sense of balance under alcohol consumption decreased. Moreover, it can be due to the increased risk of falling with limited protective reflexes.

### Limitations

The analysis presented here as well as the data published in the current literature cannot clarify sufficiently the existing relationship of the effect of ethanol in the context of an existing trauma with and without additional TBI. The discrepancy may be due to the numerous dose-dependent effects of alcohol, the time of exposure in relation to the injury and the nature of the injury and its outcome. In addition, it is very difficult to find comparable collectives in which an effect on hemodynamics and other physiological blood parameters can be investigated. Also, in the present evaluation only correlations, no causal relationships can be described. Additional studies are needed to further investigate the mechanism and possible therapeutic implications of this association.

## Conclusion

This retrospective study of the TraumaRegister DGU^®^ analyzes the influence of blood alcohol levels in seriously injured patients. The collective of alcoholic patients shows, among other things, more serious injuries in the area of the head as well as a negative influence on various physiological parameters. However, significant differences in outcome are especially apparent in alcoholic patients with an isolated TBI (AIS head ≥3). Comparable collectives need to be found and the effects of alcohol to be further investigated in clinical-experimental studies.

## Data Availability

Please contact author for data requests. Data were provided and analyzed under support of the TraumaRegister DGU® of the German Society of Trauma Surgery (DGU).

## Abbreviation

AIS: Abbreviated Injury Scale
BAL: Blood Alcohol Level
BE: Base Excess
CI: Confidence Interval
DGU: Deutsche Gesellschaft für Unfallchirurgie
GCS: Glasgow Coma Scale
ICU: Intensive Care Unit
INR: International Normalized Ratio
ISS: Injury Severity Score
NISS: New Injury Severity Score
LOS: Length of stay
TBI: Traumatic Brain Injury
